# World Rabies Day — September 28, 2014

**Published:** 2014-09-26

September 28, 2014, is the 8th annual World Rabies Day. Rabies is a fatal acute encephalitis caused by lyssaviruses (1). The number of human rabies deaths worldwide is estimated to exceed 55,000 each year (2). In the United States, wild animal reservoirs serve as the most important source of infection. However, over 90% of human deaths globally are caused by bites by rabid dogs (3).

Rabies control and prevention efforts focus on elimination of canine rabies through mass vaccination campaigns and treatment of exposed persons with prompt wound care and administration of human rabies immune globulin and vaccine. Although rabies is preventable, a lack of accurate data on the burden of disease, inadequate rabies diagnostic laboratory capacity, and poor access to rabies vaccine for postexposure prophylaxis has delayed progress towards regional goals for human rabies elimination.

Blueprints developed by international rabies experts can be used for the development of country-specific rabies elimination plans (4). These blueprints focus on describing the epidemiology of rabies, improving surveillance, raising awareness among clinicians and the public, achieving high canine vaccination coverage, and ensuring reliable diagnostic, cold chain, and vaccine procurement capacity (5).

Despite many challenges, considerable progress has been made in the Western Hemisphere; human rabies mortality has been reduced by more than 90% over the past century (6). Communicable disease programs proven to be successful in settings similar to those where canine rabies is endemic can be emulated for rabies control and prevention efforts in the future. Also, lessons learned during rabies control efforts can prove valuable for responding to emerging zoonotic diseases (7).
